# Supplementary material for: GC-Content Evolution in Bacterial Genomes: The Biased Gene Conversion Hypothesis Expands
Source: PLoS Genet. 2015 Feb 6;11(2):e1004941. doi: 10.1371/journal.pgen.1004941 (PMC4450053; doi:10.1371/journal.pgen.1004941)
Supplement: S1 Text — (DOC) [file pgen.1004941.s001.doc]

**Supplementary figures:**

**Figure S1 – Comparison of several recombination detection programs on the inferred effect of recombination on**

**core genes GC-content**

Legend as in Fig. 1.

**Figure S2 – Difference of GC% between intergenes located next to recombining vs. non−recombining genes**

Difference in average GC-content of intergenic regions around each single-copy core gene which had a conclusive result of the PHI test. Individual intergenic GC% values were computed as the average of both flanking intergenes when they were 50bp or longer, measured on at most 400bp away of the reference gene. Intergenes were classified as recombinant and non-recombinant as was the neighbouring gene based on the PHI test. A positive difference indicates that intergenes next to recombinant families are enriched in GC. Symbols and dataset abbreviations as in Fig. 1.

**Figure S3 – Comparison of several recombination detection programs on the inferred effect of recombination on**

**codon usage of core genes, based on RP optimal codons.**

Legend as in Fig. 2.

**Figure S4 – Comparison of several recombination detection programs on the inferred effect of recombination on**

**codon usage of core genes, based on HCB optimal codons from Hershberg and Petrov (2009).**

Legend as in Fig. 2.

**Supplementary Tables:**

**Table S1 – Detailed results of statistical tests of difference of GC% and Fop/Fnop between recombining and nonrecombining**

**core genes, varying recombination detection methods and tested alignment length.**

**Table S2 – Sets of optimal/non-optimal codons defined using RP method**

**Table S3 – Sets of optimal/non-optimal codons defined using HCB method**

Data derived from dataset established by Hershberg and Petrov[30].

Supplementary Text:

***Recombination detection methods***

Since most of the present work relies on relating recombination to other molecular evolution parameters, it was crucial to use an accurate method for recombination detection from gene alignments. Several methods have been developed in the last two decades, with a large spectrum of complexity in modeling and testing the presence of recombination in sequences. PHI [1] was chosen as a reference first because Bruen and colleagues compared the PHI statistics to a broad panel of other measures and statistics existing at the time of their publication: MaxChi² [2], NSS [3], measures of correlation of linkage desequilibrium (r² and |D'|) with distance [4–6] and results obtained from a coalescent-based likelihood permutation test (LPT) from LDHat [6]. In this benchmark, PHI appeared in general to be equally or more sensitive and specific than all other methods [1], and was much faster to compute than coalescent-based methods, which was a notable advantage given the size of the datasets we planned to analyze. Nonetheless, we replicated our analyses with several methods, namely PHI, NSS, MaxChi2 (as implemented in the Phipack package) and Geneconv [7]. MaxChi2 and Geneconv methods are based on the observation of unusual patterns of sequence similarity emerging from gene conversion, when NSS, like PHI, relies on the recognition of spatial patterns of phylogenetic signals along a sequence alignment. All methods gave qualitatively very similar results. We also tried two other methods not discussed in Bruen et al.: the SBP/GARD algorithm from the HyPhy package [8] and the ClonalFrame/ClonalOrigin program [9,10]. Both methods use explicit modeling of recombination in a coalescent-based framework.

GARD is a genetic algorithm using simulated populations to sample possible scenarii of sequence evolution under a model including recombination [8]. While proving very sensitive on punctual analyses, it appeared difficult to use the GARD method at large scale. As its sensitivity depends on a good sampling of the virtual populations that are simulated, one needs to take large samples to obtain robust results, which requires very intensive computation. Comparison of results from GARD and PHI showed general agreement of the methods (data not shown), so PHI was preferred for its rapidity and robustness.

ClonalOrigin is a Bayesian method for describing the history of recombination in datasets of complete genomes [10]. It has the advantage of providing quantitative estimates of the coalescent model parameters, including the locus-specific recombination rate, rather than just testing for the presence of recombination. Using this method could have allowed us to correlate the recombination rate to the GC-content of genes. We thus ran a pilot ClonalOrigin analysis on the *Spyo* dataset which had a moderate size (12 genomes, 496 core gene families of with genes of length ≥900bp) and an expected strong relation between recombination and GC-content, given the previous analysis based on PHI test (see Main text and Fig. 1). The computation time necessary to finish ClonalOrigin inference is of several weeks on one CPU per gene family alignment, which would amount to more than a thousand years computation time to cover all our datasets if run sequentially. Even with the possibility of parallelization, this seemed prohibitive and led us to prefer PHI statistics and to perform pairwise comparison tests of GC% in recombining vs. non-recombining genes rather than correlations for the global analysis of our 21 datasets. Nonetheless, we were able to verify the agreement of results from ClonalOrigin and PHI and assess the predictive power of the estimated recombination rate on the GC-content for the *Spyo* dataset. Gene families detected as recombinant by PHI had a significantly higher mean recombination rate (*rho*) than non-recombinant ones (76.6 vs. 50.1, Student's *t*-test *p* < 10^-4^). Correlations of *rho* estimate with GC% or GC3 were significant though very low (*r*^2^ = 2.5%, *p* < 10^-3^ and *r*^2^ = 3.4%, *p* < 10^-4^, respectively). However, we observed difficulties to reach convergence when estimating the *rho* parameter, especially with gene alignments where the average estimated recombination frequency was high (data not shown). ClonalOrigin highly-parameterized model seems more appropriate to work on large syntenic blocks of genomes than on gene alignments. Indeed, some studies reported “biases from boundary effects in short alignment blocks” when working on alignments shorter than 1.5kb [11]. Inaccurate estimates of *rho* may thus account for the weak observed correlation. Indeed, when excluding families for which the rho estimate had a high variance along the MCMC sample (42/478), the correlation with GC% or GC3 raised to *r*^2^ = 8.4% (*p* < 10^-9^) and *r*^2^ = 8.7% (*p* < 10^-9^), respectively.

*References*

1. Bruen TC, Philippe H, Bryant D (2006) A Simple and Robust Statistical Test for Detecting the Presence of Recombination. Genetics 172: 2665–2681. doi:10.1534/genetics.105.048975.

2. Smith JM (1992) Analyzing the mosaic structure of genes. J Mol Evol 34: 126–129.

3. Jakobsen IB, Easteal S (1996) A program for calculating and displaying compatibility matrices as an aid in determining reticulate evolution in molecular sequences. Comput Appl Biosci CABIOS 12: 291–295.

4. Hill WG, Robertson A (1968) Linkage disequilibrium in finite populations. Theor Appl Genet: 54–78.

5. Miyashita N, Langley CH (1988) Molecular and phenotypic variation of the white locus region in Drosophila melanogaster. Genetics 120: 199–212.

6. McVean G, Awadalla P, Fearnhead P (2002) A coalescent-based method for detecting and estimating recombination from gene sequences. Genetics 160: 1231–1241.

7. Sawyer S (1989) Statistical tests for detecting gene conversion. Mol Biol Evol 6: 526–538.

8. Pond SLK, Posada D, Gravenor MB, Woelk CH, Frost SDW (2006) Automated Phylogenetic Detection of Recombination Using a Genetic Algorithm. Mol Biol Evol 23: 1891–1901. doi:10.1093/molbev/msl051.

9. Didelot X, Falush D (2007) Inference of bacterial microevolution using multilocus sequence data. Genetics 175: 1251–1266. doi:10.1534/genetics.106.063305.

10. Didelot X, Lawson D, Darling A, Falush D (2010) Inference of Homologous Recombination in Bacteria Using Whole Genome Sequences. Genetics 186: 1435–1449. doi:10.1534/genetics.110.120121.

11. Choi SC, Rasmussen MD, Hubisz MJ, Gronau I, Stanhope MJ (2012) Replacing and Additive Horizontal Gene Transfer in Streptococcus. Mol Biol Evol. 29(11):3309-20. doi: 10.1093/molbev/mss138.
